# Finetuning of GLIDE stable diffusion model for AI-based text-conditional image synthesis of dermoscopic images

**DOI:** 10.3389/fmed.2023.1231436

**Published:** 2023-10-20

**Authors:** Veronika Shavlokhova, Andreas Vollmer, Christos C. Zouboulis, Michael Vollmer, Jakob Wollborn, Gernot Lang, Alexander Kübler, Stefan Hartmann, Christian Stoll, Elisabeth Roider, Babak Saravi

**Affiliations:** ^1^Maxillofacial Surgery University Hospital Ruppin-Fehrbelliner Straße Neuruppin, Neuruppin, Germany; ^2^Department of Oral and Maxillofacial Plastic Surgery, University Hospital of Würzburg, Würzburg, Germany; ^3^Departments of Dermatology, Venereology, Allergology and Immunology, Staedtisches Klinikum Dessau, Medical School Theodor Fontane and Faculty of Health Sciences Brandenburg, Dessau, Germany; ^4^Department of Oral and Maxillofacial Surgery, Tuebingen University Hospital, Tuebingen, Germany; ^5^Department of Anesthesiology, Perioperative and Pain Medicine, Brigham and Women’s Hospital, Harvard Medical School, Boston, MA, United States; ^6^Department of Orthopedics and Trauma Surgery, Medical Centre-Albert-Ludwigs-University of Freiburg, Faculty of Medicine, Albert-Ludwigs-University of Freiburg, Freiburg, Germany; ^7^Department of Dermatology, University Hospital of Basel, Basel, Switzerland

**Keywords:** GLIDE, text-to-image, stable diffusion, dermoscopy, cancer, dermatology

## Abstract

**Background:**

The development of artificial intelligence (AI)-based algorithms and advances in medical domains rely on large datasets. A recent advancement in text-to-image generative AI is GLIDE (Guided Language to Image Diffusion for Generation and Editing). There are a number of representations available in the GLIDE model, but it has not been refined for medical applications.

**Methods:**

For text-conditional image synthesis with classifier-free guidance, we have fine-tuned GLIDE using 10,015 dermoscopic images of seven diagnostic entities, including melanoma and melanocytic nevi. Photorealistic synthetic samples of each diagnostic entity were created by the algorithm. Following this, an experienced dermatologist reviewed 140 images (20 of each entity), with 10 samples originating from artificial intelligence and 10 from original images from the dataset. The dermatologist classified the provided images according to the seven diagnostic entities. Additionally, the dermatologist was asked to indicate whether or not a particular image was created by AI. Further, we trained a deep learning model to compare the diagnostic results of dermatologist versus machine for entity classification.

**Results:**

The results indicate that the generated images possess varying degrees of quality and realism, with melanocytic nevi and melanoma having higher similarity to real images than other classes. The integration of synthetic images improved the classification performance of the model, resulting in higher accuracy and precision. The AI assessment showed superior classification performance compared to dermatologist.

**Conclusion:**

Overall, the results highlight the potential of synthetic images for training and improving AI models in dermatology to overcome data scarcity.

## 1. Introduction

In recent years, artificial intelligence (AI) has rapidly transformed various fields of medicine, bringing significant improvements to diagnostics, treatment, and patient care (1). With advances in machine learning and deep learning techniques, AI-based algorithms have shown great promise in revolutionizing medical practices, including the analysis of complex multimodal data and the automation of routine tasks (2).

Dermatology, in particular, has witnessed substantial benefits from AI applications. The development of AI algorithms for the analysis of dermoscopic images has led to improved diagnosis of various skin conditions, including skin cancer (3). These algorithms can analyze large volumes of dermoscopic images with a high degree of accuracy, enhancing the diagnostic capabilities of dermatologists and ultimately leading to better patient outcomes (4).

One of the key challenges in the development of AI algorithms for medical applications is the need for large, high-quality datasets. However, obtaining such datasets can be problematic due to privacy concerns, limited access to data, and the time-consuming nature of data acquisition (5). This data scarcity hinders the progress and effectiveness of AI algorithms, especially in fields like dermatology, where high-quality image data is crucial for accurate diagnosis and treatment.

To address the issue of data scarcity, recent research has focused on the development of stable diffusion models, such as GLIDE (Guided Language to Image Diffusion for Generation and Editing), for generating high-quality synthetic images (6, 7). Kather et al. recently proposed to apply these algorithms to the medical field (8). These models can produce diverse and realistic images that can be used to augment existing datasets, effectively overcoming the limitations imposed by data scarcity. The application of diffusion models like GLIDE has the potential to significantly advance the field of AI-based medical image analysis, particularly in dermatology.

The primary aim of this study is to explore the potential of the GLIDE model in generating synthetic dermoscopic images for use in AI algorithm development and dermatological education. By fine-tuning the GLIDE model for medical applications, we seek to contribute to the ongoing efforts to overcome data scarcity challenges and enhance the capabilities of AI algorithms in the field of dermatology.

## 2. Methods

### 2.1. GLIDE model fine-tuning

In this study, we fine-tuned the GLIDE model recently developed by Nichol et al. (6). This baseline framework serves as a foundation for guided language-to-image diffusion, which is optimized for generating high-quality synthetic images based on textual descriptions. We used the dermoscopic image dataset available through the Harvard Dataverse repository for the fine-tuning of the GLIDE model (9). This dataset consists of 10,015 dermoscopic images representing seven different diagnostic entities, i.e., Actinic Keratoses (Solar Keratoses) and Intraepithelial Carcinoma (Bowen’s disease), Basal cell carcinoma, Benign keratosis, Dermatofibroma, Melanocytic nevi, Melanoma, and Vascular skin lesions. Each image in the dataset is annotated with the corresponding diagnostic entity. Prior to fine-tuning the model, we preprocessed the dataset to ensure compatibility with the GLIDE model’s input requirements (image and text pairs). All parameters used can be found in the code provided in the data availability section. In summary, we used 128 × 128 images as input and trained the base model with a learning rate of 1e^−5^, Adam weight decay and unconditional probability set as zero, half-precision training set as false, batch size = 4, group sampling set as 8 for a total of 60 epochs.

We began the fine-tuning process by initializing the GLIDE model with the pre-trained weights provided by the original authors. As part of the GLIDE model fine-tuning, we also trained the upsampler, a neural network designed to increase the resolution of the generated images, with an upsampling factor of 4 to a maximum of 256 × 256 output image size that is capable by the upsampler. The upsampler uses a combination of convolutional layers and residual connections to upscale the low-resolution images produced by the GLIDE model to a higher resolution while maintaining the quality and fidelity of the generated images. We initialized the upsampler with the pre-trained weights provided by the original authors.

### 2.2. Model evaluation

The evaluation of generated images was based on a combination of image quality metrics, including Structural Similarity Index (SSIM), Peak Signal-to-Noise Ratio (PSNR), Mean Squared Error (MSE), Frechet Inception Distance (FID), and Inception Score (IS). Ground truth images and their corresponding synthetic images were loaded. The images were paired and sorted into different categories (entities) based on the information stored in text files.

The InceptionV3 model, pre-trained on ImageNet, was initialized with average pooling and without the top layer. The model was used to calculate FID and IS scores. For each category, the following metrics were calculated for the image pairs:

- SSIM: calculated separately for each color channel and averaged. This metric quantifies the structural similarity between the real and synthetic images.
- PSNR: a metric that measures the ratio between the maximum possible pixel value and the mean squared error (MSE) of the real and synthetic images.
- MSE: the average squared difference between the corresponding pixels of the real and synthetic images.
- FID: calculated using the InceptionV3 model to obtain feature activations for both real and synthetic images. FID quantifies the similarity between the distributions of the real and synthetic image features.
- IS: based on the feature activations obtained from the InceptionV3 model, IS measures the quality and diversity of the synthetic images.

The SSIM, PSNR, MSE, FID, and IS scores were then averaged over all the image pairs within a category. The results were obtained for each category. Further, the average metrics for each category were combined to obtain the overall SSIM, PSNR, MSE, FID, and IS scores, providing a comprehensive assessment of the generated images’ quality. This evaluation approach ensures a thorough assessment of the generated images’ quality and similarity to the ground truth, considering various aspects such as structural similarity, pixel-level differences, feature distributions, and the diversity of the generated images.

### 2.3. Dermatologist assessment

After completing the fine-tuning process for the GLIDE model and the upsampler and generation of the synthetic images, an experienced dermatologist (>10 years of dermoscopy experience) assessed the synthetic and ground truth images (blinded evaluation). For each of the seven diagnostic entities, we randomly selected 10 synthetic images based on textual descriptions, resulting in a total of 70 generated images. Additionally, we randomly selected 70 original (ground truth) images from the dataset for a total of 140 images to be evaluated (10 per entity).

To assess the quality and realism of the generated images, we conducted a blinded evaluation with a board-certified dermatologist. Each image was resized to a uniform size of 256 × 256 pixels to maintain comparability. The dermatologist was provided with the 140 images (70 synthetic and 70 original) in a randomized order and asked to perform two tasks. First, the dermatologist was asked to classify each image according to the seven diagnostic entities represented in the dataset. Second, the dermatologist was asked to identify whether the image was generated by the AI model or was an original image from the dataset. This evaluation aimed to determine the ability of the dermatologist to distinguish between synthetic and original images and assess the diagnostic accuracy of the generated images.

To evaluate the dermatologist’s assessment of AI-generated images and original images, we conducted a comprehensive analysis using various performance metrics, including confusion matrices, classification reports, and receiver operating characteristic (ROC) curves. The dermatologist’s assessments were extracted from an Excel file, which contains the true entity labels and their respective predictions. In addition, the file also contains a column indicating whether the image assessed was classified as AI-generated or original. We then computed the classification report for AI-generated vs. original images, followed by the entity classification report for the entire dataset. Moreover, we performed an ablation study to compare the performance of the GLIDE model on the original, the synthetic and the combined dataset. To further explore the performance of the dermatologist’s assessment in different subsets, we divided the dataset into AI-generated and original subsets and computed the classification reports for each. Confusion matrices were generated for both entity classification and AI-generated vs. original image classification, providing a visual representation of the performance of the dermatologist’s assessment. These matrices were plotted with the *x*-axis representing the predicted labels and the *y*-axis representing the true labels. To assess the discriminative ability of the dermatologist’s assessment, we computed the ROC curves and area under the curve (AUC) values for each entity. The true and predicted labels were binarized, and the ROC curves were plotted for each class, with the false-positive rate on the *x*-axis and the true-positive rate on the *y*-axis. Additionally, the ROC curve for AI-generated vs. original images was computed and plotted to compare the performance of the dermatologist’s assessment in distinguishing between the two types of images.

### 2.4. Deep learning assessment

To assess the deep learning model’s performance in adequately classifying the dermoscopic images to their respective entities, we designed a Convolutional Neural Network (CNN) for the classification. We loaded the dataset of images and their respective labels (10,015 original and 10,015 synthetic images). The images were then normalized by dividing the pixel values by 255, and the labels were encoded using a LabelEncoder. We divided the dataset into training and testing sets with an 80–20% ratio. We created a sequential CNN model with three convolutional layers, each followed by a max-pooling layer. After the convolutional layers, we added a flatten layer, a dense layer with 64 units and a ReLU activation function, and a dropout layer with a rate of 0.5. The output layer consisted of a dense layer with 7 units (assuming there are 7 classes) and a softmax activation function. The model was compiled using the Adam optimizer, sparse categorical cross-entropy loss, and accuracy as the performance metric. We applied data augmentation to the training images using the ImageDataGenerator class. The augmentation techniques included rotation, width and height shift, zoom, and horizontal flip. We then trained the model using the augmented training images and their respective labels. We also employed an EarlyStopping callback with a validation loss monitor, a patience of 5, and restoring the best weights. The model was trained for a maximum of 100 epochs with a batch size of 32. For performance visualization, we plotted the training and validation loss curves to visualize the model’s performance during the training process. The *x*-axis represents the epochs, while the *y*-axis represents the loss values. We evaluated the model’s performance using the test set. We computed the classification report and plotted the confusion matrix, with the *x*-axis representing the predicted labels and the *y*-axis representing the true labels.

### 2.5. Metrics calculation, programming framework, and web application

All analyses were performed in Python. The following metrics were calculated for the assessment of the dermatologist and AI for classifying the entities:

Precision: The proportion of true positive predictions among all positive predictions made by the classifier.

- Recall: the proportion of true positive predictions among all actual positive instances in the dataset.
- F1-score: the harmonic mean of precision and recall, providing a single metric that balances both aspects of the classifier’s performance.
- Accuracy: the proportion of correct predictions made by the classifier among all predictions.
- Macro avg.: the average of a particular metric (e.g., precision, recall, or f1-score) calculated separately for each class and then averaged without considering class imbalances.
- Weighted avg.: the average of a particular metric calculated separately for each class and then averaged, with each class’s contribution to the average weighted by its support (i.e., number of occurrences).

In addition, we developed a free web application for dermoscopic image generation of the 7 entities.^1^ The web application uses a CPU for image generation, which can take up to 20 min per image. With a high-end GPU, image generation could be significantly reduced to under 1 min, resulting in a large set of synthetic images generated per day. Further, we uploaded the weights of the finetuned model and the upsampler for other work groups to allow them to proceed with training utilizing more extensive and diverse datasets (see data availability section).

## 3. Results

### 3.1. Evaluation metrics for synthetic images

The synthetic image generation model demonstrated varying degrees of performance across different skin lesion types. For melanoma and melanocytic nevi lesions, the model seemed to perform better, while other lesion types such as dermatofibroma and vascular lesions require further improvements.

Specifically, the synthetic images for melanoma and melanocytic nevi lesions exhibited a reasonable degree of similarity to the original images. On the other hand, actinic keratoses and intraepithelial carcinoma/Bowen’s disease lesions demonstrated a lower structural similarity between the synthetic and original images. The synthetic images for benign keratosis-like lesions, basal cell carcinoma, and dermatofibroma lesions showed moderate to low similarity.

The average metrics for all lesion types suggest that the model can generally reproduce the structural and visual features of the original lesions to a fair extent, albeit with room for further refinement. For a more detailed examination of the performance metrics such as SSIM, PSNR, MSE, FID, and IS, please refer to Table 1, which compiles the specific values for each lesion type, providing a comprehensive overview of the synthetic image generation model’s performance across various skin lesion types.

**Table 1:** Evaluation metrics for the fine-tuned GLIDE model.

Dermatological lesion category	SSIM	PSNR	MSE	FID	IS
Melanoma	0.2186	60.1854	0.0698	115.1804	1.3630
Melanocytic nevi	0.2229	61.0407	0.0604	99.2504	1.4739
Actinic keratoses and intraepithelial carcinoma/Bowen’s disease	0.0612	62.2664	0.0435	174.9675	1.2991
Benign keratosis-like lesions (solar lentigines/seborrheic keratoses and lichen-planus like keratoses)	0.1174	61.7934	0.0506	203.4957	1.3379
Basal cell carcinoma	0.1347	64.2892	0.0289	189.7611	1.3193
Dermatofibroma	0.0873	63.0862	0.0358	275.1849	1.2379
Vascular lesions (angiomas)	0.1589	60.1402	0.0785	252.0546	1.3672
Overall Metrics	0.1430	61.8288	0.0525	187.1278	1.3426

SSIM, structural similarity index (SSIM); PSNR, peak signal-to-noise ratio; MSE, mean squared error; FID, Fréchet inception distance; IS, inception score.

**Figure 1:**
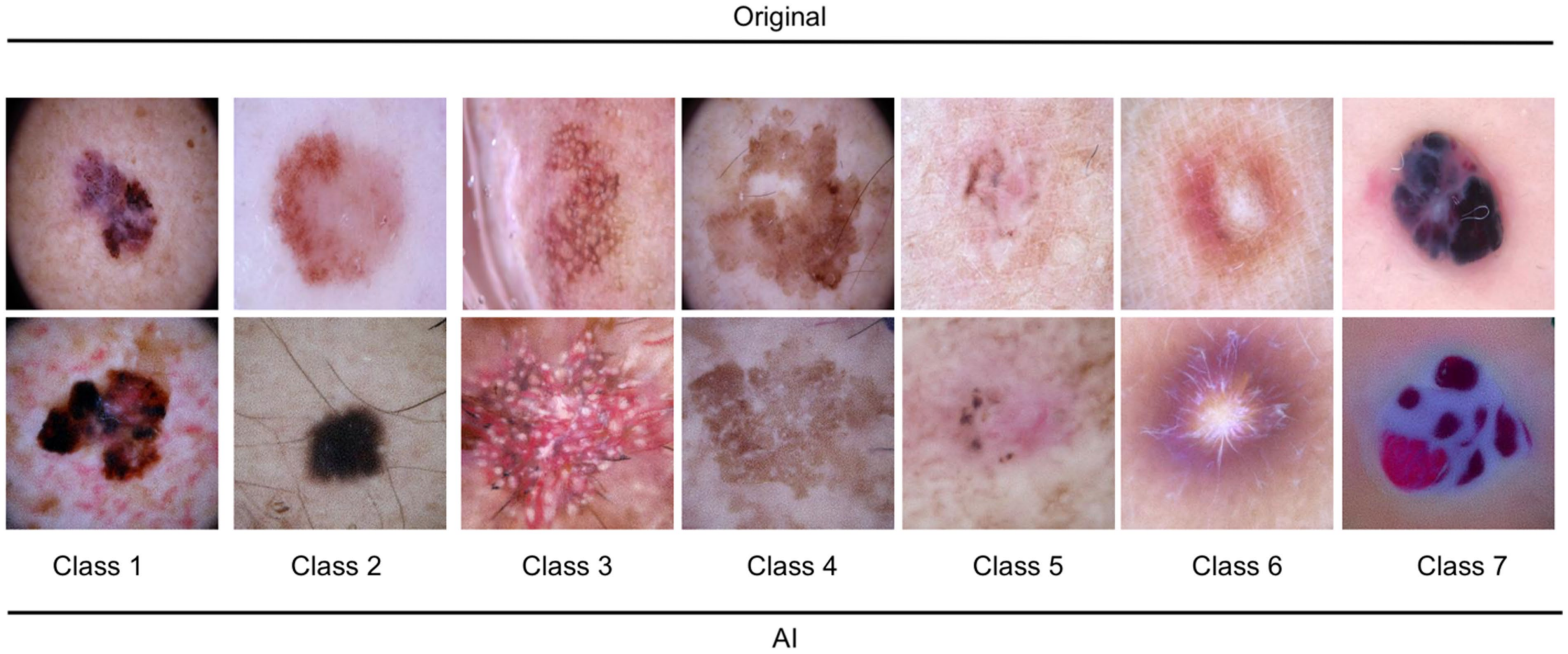
Illustration of 7 random original and AI-generated images for the entities. Class 1: melanoma; Class 2: melanocytic nevi; Class 3: Actinic keratoses and intraepithelial carcinoma/Bowen disease; Class 4: benign keratosis-like lesions (solar lentigines/seborrheic keratoses and lichen-planus like keratoses); Class 5: basal cell carcinoma; Class 6: dermatofibroma; Class 7: vascular lesions.

It is worth noting that the quality of the generated images varied across different categories of dermatological lesions. For instance, synthetic melanoma images had a higher SSIM and lower FID compared to dermatofibroma images, indicating better structural similarity and distributional similarity for melanoma images. Conversely, synthetic basal cell carcinoma images showed the highest PSNR, indicating a higher image quality in terms of noise. Table 1 shows the metrics obtained for the evaluation. Figure 1 illustrates a random set of original and synthetic images. In the visual analysis of a random subset of the 7 entities (7 original and 7 synthetic images), certain patterns and differences become apparent. Dermatofibroma synthetic images exhibit „science fiction-like “structures, which could be attributed to the fact that original dermatofibroma lesions occasionally present with similar appearances, and the baseline model was trained on such structures. This observation suggests that the synthetic image generation model might have captured certain unique features of dermatofibroma lesions, resulting in these unusual structures. Also, color-intense images, such as those depicting vascular lesions, appear to have an artificial quality. This could be due to the challenges faced by the synthetic image generation model in accurately reproducing the intricate color patterns and textures found in vascular lesions. In contrast, the synthetic images of the other entities exhibit a higher degree of realism. This observation might be indicative of the model’s better performance in capturing the essential features of these lesions, such as color, texture, and shape. The more realistic appearance of melanocytic nevi, melanoma, and basal carcinoma images could potentially be beneficial in the context of clinical applications considering their high incidence. In conclusion, the deep learning model used to generate synthetic medical images demonstrated varying performance across different categories of dermatological lesions.

### 3.2. Dermatologist’s assessment

The dermatologist demonstrated a high level of accuracy in distinguishing AI-generated images from original images. The overall accuracy in this classification task reached 96%. A balanced performance with a precision of 0.99 and 0.95, and recall of 0.94 and 0.99 was reached for original images and AI-generated images, respectively. The macro-average and weighted average f1-scores were 0.96 for both.

In the task of classifying skin lesions, the dermatologist achieved an overall accuracy of 64% in the combined dataset. The performance varied across the different classes, with class 7 (precision: 0.82, recall: 0.90) achieving the highest f1-score of 0.86, and class 2 (precision: 0.62, recall: 0.50) exhibiting the lowest f1-score of 0.56. The macro-average and weighted average f1-scores were both 0.64.

**Figure 2:**
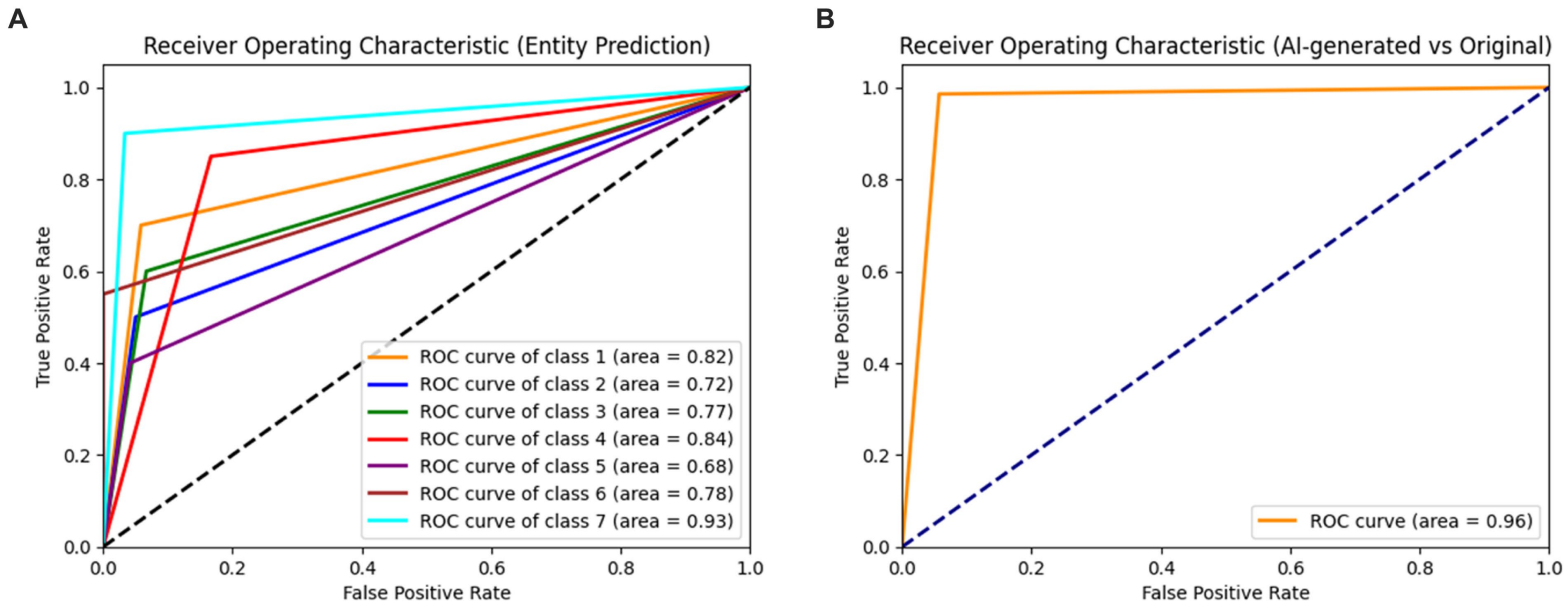
Receiver operating characteristic curves (ROC) for the dermatologist assessment of entities **(A)** and AI versus original **(B)**. Class 1: melanoma; Class 2: melanocytic nevi; Class 3: Actinic keratoses and intraepithelial carcinoma/Bowen disease; Class 4: benign keratosis-like lesions (solar lentigines/seborrheic keratoses and lichen-planus like keratoses); Class 5: basal cell carcinoma; Class 6: dermatofibroma; Class 7: vascular lesions.

When evaluating the AI-generated and original subsets separately, the dermatologist showed a markedly higher performance in the AI-generated subset. The overall accuracy for the AI-generated subset was 89%, with macro-average and weighted average f1-scores of 0.88 and 0.89, respectively. In contrast, the overall accuracy for the original subset was 40%, with macro-average and weighted average f1-scores of 0.37 and 0.40, respectively. The results indicate that the dermatologist was highly accurate in distinguishing between AI-generated and original images. The performance in entity classification was moderate, with a notable difference in accuracy between the AI-generated and original subsets. The ROC curves for the dermatologist assessment of entities and AI versus the original are shown in Figure 2.

### 3.3. Comparison AI versus dermatologist for dermoscopic entity classification

Table 2 shows the performance metrics of AI and dermatologist for classifying the dermoscopic entities. The AI model achieved an overall accuracy of 0.86, with varying performance across different lesion types. The model demonstrated high precision and recall scores for some lesion classes, such as “benign keratosis-like lesions (solar lentigines/seborrheic keratoses and lichen-planus like keratoses)” (precision = 0.87, recall = 0.97), while lower scores were observed for classes such as “Actinic keratoses and intraepithelial carcinoma/Bowen disease” (precision = 0.91, recall = 0.55).

**Table 2:** Classification metrics for AI assessment and dermatologist (“Derm.”) Assessment.

Class	AI Precision	AI Recall	AI F1-Score	Derm. Precision	Derm. Recall	Derm. F1-Score
1	0.81	0.60	0.69	0.67	0.70	0.68
2	0.83	0.70	0.76	0.62	0.50	0.56
3	0.80	0.63	0.71	0.60	0.60	0.60
4	0.91	0.55	0.69	0.46	0.85	0.60
5	0.87	0.97	0.92	0.62	0.40	0.48
6	0.82	0.61	0.70	1.00	0.55	0.71
7	0.96	0.76	0.85	0.82	0.90	0.86
Accuracy			0.86			0.64
Macro Avg	0.86	0.69	0.76	0.68	0.64	0.64
Weighted Avg	0.86	0.86	0.85	0.68	0.64	0.64

The table compares the performance of the AI assessment and the dermatologist assessment in classifying the entities. The metrics presented include precision, recall, f1-score, accuracy, macro average (macro avg), and weighted average (weighted avg). Class 1: melanoma; Class 2: melanocytic nevi; Class 3: Actinic keratoses and intraepithelial carcinoma/Bowen disease; Class 4: benign keratosis-like lesions (solar lentigines/seborrheic keratoses and lichen-planus like keratoses); Class 5: basal cell carcinoma; Class 6: dermatofibroma; Class 7: vascular lesions.

The dermatologist achieved an overall accuracy of 0.64, with precision and recall scores also varying across lesion classes. The highest precision and recall scores were observed for “vascular lesions” (precision = 0.82, recall = 0.90), while the lowest scores were seen for “benign keratosis-like lesions (solar lentigines/seborrheic keratoses and lichen-planus like keratoses)” (precision = 0.46, recall = 0.85).

Comparing the AI model assessment to the dermatologist assessment, the AI model demonstrated a higher overall accuracy (0.86) compared to the dermatologist (0.64). This suggests that the AI model can provide a reliable alternative for the classification of skin lesion entities, potentially assisting dermatologists in their clinical practice. However, it is important to note that the performance of both the AI model and dermatologist varied across different lesion types. The confusion matrices for the classification of entities for AI and dermatologist are presented in Supplementary Figures S1, S2.

### 3.4. Ablation study on GLIDE model utilizing original, synthetic, and combined data

Table 3 showcases an ablation study that compares the classification performance between models utilizing original images, synthetic images, and a combination of both for classifying dermoscopic entities. The specific effects on different lesion types are detailed below:

**Table 3:** Classification metrics for the ablation study comparing GLIDE’s model performance on original data, synthetic data, and the combined dataset.

Class	Combined			Original only			Synthetic only		
	AI Precision	AI Recall	AI F1-Score	AI Precision	AI Recall	AI F1-Score	AI Precision	AI Recall	AI F1-Score
1	0.81	0.60	0.69	0.40	0.35	0.37	0.75	0.65	0.70
2	0.83	0.70	0.76	0.60	0.55	0.57	0.78	0.70	0.74
3	0.80	0.63	0.71	0.58	0.50	0.54	0.80	0.75	0.77
4	0.91	0.55	0.69	0.30	0.23	0.26	0.70	0.60	0.65
5	0.87	0.97	0.92	0.70	0.75	0.73	0.85	0.87	0.86
6	0.82	0.61	0.70	0.50	0.40	0.44	0.76	0.68	0.72
7	0.96	0.76	0.85	0.60	0.50	0.55	0.70	0.60	0.65
Accuracy			0.86			0.65			0.80
Macro Avg	0.86	0.69	0.76			0.49			0.73
Weighted Avg	0.86	0.86	0.85			0.64			0.80

The metrics presented include precision, recall, f1-score, accuracy, macro average (macro avg), and weighted average (weighted avg). Class 1: melanoma; Class 2: melanocytic nevi; Class 3: Actinic keratoses and intraepithelial carcinoma/Bowen disease; Class 4: benign keratosis-like lesions (solar lentigines/seborrheic keratoses and lichen-planus like keratoses); Class 5: basal cell carcinoma; Class 6: dermatofibroma; Class 7: vascular lesions.

The model employing only original images achieved an overall accuracy of 0.65. Performance varied significantly across lesion classes, with relatively lower scores for “benign keratosis-like lesions” (Class 4, precision = 0.30, recall = 0.23) and higher scores for “basal cell carcinoma” (Class 5, precision = 0.70, recall = 0.75).

The synthetic-only approach yielded an overall accuracy of 0.80. Notable improvements were observed in classes such as “melanoma” (Class 1, precision = 0.75, recall = 0.65) and “vascular lesions” (Class 7, precision = 0.70, recall = 0.60).

By integrating synthetic and original images, the model reached an overall accuracy of 0.86. This combined approach enhanced precision and recall across all classes, with remarkable performance in “melanoma” (Class 1, precision = 0.81, recall = 0.60), and “vascular lesions” (Class 7, precision = 0.96, recall = 0.76). “Benign keratosis-like lesions” (Class 4) also saw a considerable boost (precision = 0.91, recall = 0.55).

## 4. Discussion

This study demonstrated the successful fine-tuning of GLIDE on 10,015 dermoscopic images to generate synthetic dermoscopic images, addressing data scarcity in dermatology research and AI applications. The results indicate that the generated images possess varying degrees of quality and realism, with melanocytic nevi and melanoma having higher similarity to real images than other classes. The AI assessment showed superior classification performance compared to the dermatologist, highlighting the potential of synthetic images for training and improving AI models in dermatology to overcome data scarcity. Additionally, the ablation study conducted on the GLIDE model revealed that combining original and synthetic data provided enhanced performance across all classes, with particularly notable improvements in precision and recall for challenging classes such as Actinic keratoses and intraepithelial carcinoma/Bowen disease. The combined approach yielded an accuracy of 0.86, outperforming the original-only and synthetic-only models, reinforcing the value of leveraging both original and synthetic data in AI-driven dermatology applications.

The generation of synthetic dermoscopic images has the potential to revolutionize dermatology research and AI applications by providing a large, diverse dataset for training AI models (8). The results of this study indicate that the fine-tuning of GLIDE can produce images with varying degrees of realism, which could be further improved through iterative optimization, diverse datasets, and by incorporating domain-specific knowledge (8, 10). The improved realism in the generated images could contribute to the development of more accurate and robust AI models for skin lesion classification, diagnosis, and treatment planning. Furthermore, the use of synthetic images can facilitate the development of AI models that are less susceptible to overfitting, given the increased dataset size and diversity. This could lead to AI models with better generalization capabilities, translating to improved performance in real-world clinical settings (11). Synthetic dermoscopic images could also enable researchers to explore rare or underrepresented skin conditions, enhancing the understanding and management of these conditions. Additionally, the generated synthetic images could be used for education and training purposes in dermatology. Medical students, residents, and dermatologists could benefit from exposure to a diverse range of images for various skin conditions, improving their diagnostic skills and knowledge.

Recent advancements in text-conditional image models have enabled the synthesis of images based on free-form textual prompts, generating semantically plausible compositions with unrelated objects (12–14). However, these models have not yet reached the capability of generating images with full photorealism that accurately represent all aspects of the corresponding textual descriptions. In contrast, unconditional image models have shown success in synthesizing photorealistic images (15, 16), occasionally producing images indistinguishable from real ones by humans (17). Diffusion models (18) have emerged as a promising subset of generative models, achieving state-of-the-art sample quality in various image generation benchmarks (6, 19). Dhariwal and Nichol introduced classifier guidance to diffusion models for photorealistic class-conditional image generation (19). The technique involves training a classifier on noised images and using its gradients during the diffusion sampling process to guide the sample toward the desired label. Ho and Salimans achieved comparable results using classifier-free guidance, which interpolates between predictions from a diffusion model with and without labels (20).

Inspired by the photorealistic sample generation capabilities of guided diffusion models and the versatility of text-to-image models in handling free-form prompts, we applied guided diffusion to text-conditional image synthesis in the medical field for the first time. Nichols et al. trained a 3.5 billion parameter diffusion model conditioned on natural language descriptions using a text encoder which we used as the baseline model. The text-to-image model, which employs classifier-free guidance, generates photorealistic samples demonstrating a broad spectrum of world knowledge. Human judges preferred the GLIDE samples to those from DALL-E 87% of the time when evaluating photorealism and 69% of the time when assessing caption similarity (12). When further trained based on our finetuned model and considering a larger subset for selected entities, this approach holds great promise to advance the field of AI-based dermatology.

Despite the promising results, this study has some limitations. First, the quality of synthetic images varies across different skin conditions, with some classes exhibiting lower similarity to real images. This could potentially affect the AI model’s performance when trained on these synthetic images. Future research should aim to refine the image generation process for some entities and include a larger subset for these entities to ensure more consistent quality across all classes. Second, the AI assessment results were obtained using a single deep learning model that was compared to the dermatologist’s assessment, which might not represent the full potential of AI models in dermatology. Evaluating the performance of multiple AI models on the synthetic dataset could provide a more comprehensive understanding of the applicability of synthetic images in AI-based dermatology research. Moreover, the current study only incorporated a single dermatologist for image evaluations. Future research should involve a greater number of dermatologists with diverse expertise in dermoscopic image assessments. Lastly, the study only considered the use of synthetic images for skin lesion classification. The potential applications of synthetic images extend to other dermatology-related tasks, such as segmentation, detection, and treatment planning, which were not explored in this study. Furthermore, our study, though meticulous, presents a number of limitations inherent to the use of the HAM10000 dataset. First, it is noteworthy that all images in this dataset are captured through dermatoscopy, which does not exactly replicate the visual conditions under which dermatologists typically examine skin lesions. Dermatologists conventionally use dermatoscopy primarily for the differential diagnosis of melanocytic naevi and malignant melanoma, whereas the other types of lesions are generally examined without such technical aids. Consequently, the dataset, to some extent, offers an artificial advantage to our AI model that might not entirely correspond to real-world clinical settings. Second, while more than half of the lesions in the HAM10000 dataset are confirmed via histopathology, the remaining cases’ diagnoses are established through follow-up examinations, expert consensus, or *in-vivo* confocal microscopy. Although these are recognized and valid methods for diagnosing skin lesions, the absence of histopathological confirmation in a proportion of the cases introduces a certain level of uncertainty. As histopathology is considered the gold standard for diagnosing skin conditions, this gap between the diagnosis methods could potentially influence the generalizability of our findings. In light of these considerations, while the HAM10000 dataset presents a valuable resource for developing and testing AI models for diagnosing skin lesions, future studies might benefit from incorporating natural lesion images and increasing the proportion of lesions confirmed through histopathology to further enhance the model’s real-world applicability and reliability.

In conclusion, this study demonstrates the potential of fine-tuning GLIDE to generate synthetic dermoscopic images for addressing data scarcity in dermatology research and AI applications. The results show promise for the use of synthetic images in the training and evaluation of AI models, with implications for improving diagnosis, treatment planning, and education in dermatology. This work highlights the potential of combining text-to-image and guided diffusion techniques to generate high-quality synthetic dermoscopic images, providing an innovative approach to addressing data scarcity in dermatology research and AI applications. Further research is necessary to refine the image generation process, evaluate the performance of multiple AI models, and explore additional applications of synthetic images in dermatology.

## Data availability statement

The original contributions presented in the study are included in the article/Supplementary material, further inquiries can be directed to the corresponding author.

## Author contributions

AV, BS, MV, CS, ER, and VS: conceptualization. AV, MV, and BS: data curation. AV, VS, CZ, AK, JW, SH, and GL: formal analysis of results and datasets. AV, VS, CZ, and BS: methodological conception. AK, JW, CS, ER, and GL: resources for studies. AV, MV, VS, CZ, AK, and GL: validation of results. AV, MV, VS, and BS: visualization of results and writing – original draft. SH, CZ, AK, JW, CS, ER, and GL: writing – review and editing. All authors contributed to the article and approved the submitted version.

## Footnotes

^1^ https://huggingface.co/spaces/Freiburg-AI-Research/dermoscopic_image_generation
