# Supplementary figures and images for: Finetuning of GLIDE stable diffusion model for AI-based text-conditional image synthesis of dermoscopic images
Source: Front Med (Lausanne). 2023 Oct 20;10:1231436. doi: 10.3389/fmed.2023.1231436 (PMC10623307; doi:10.3389/fmed.2023.1231436)

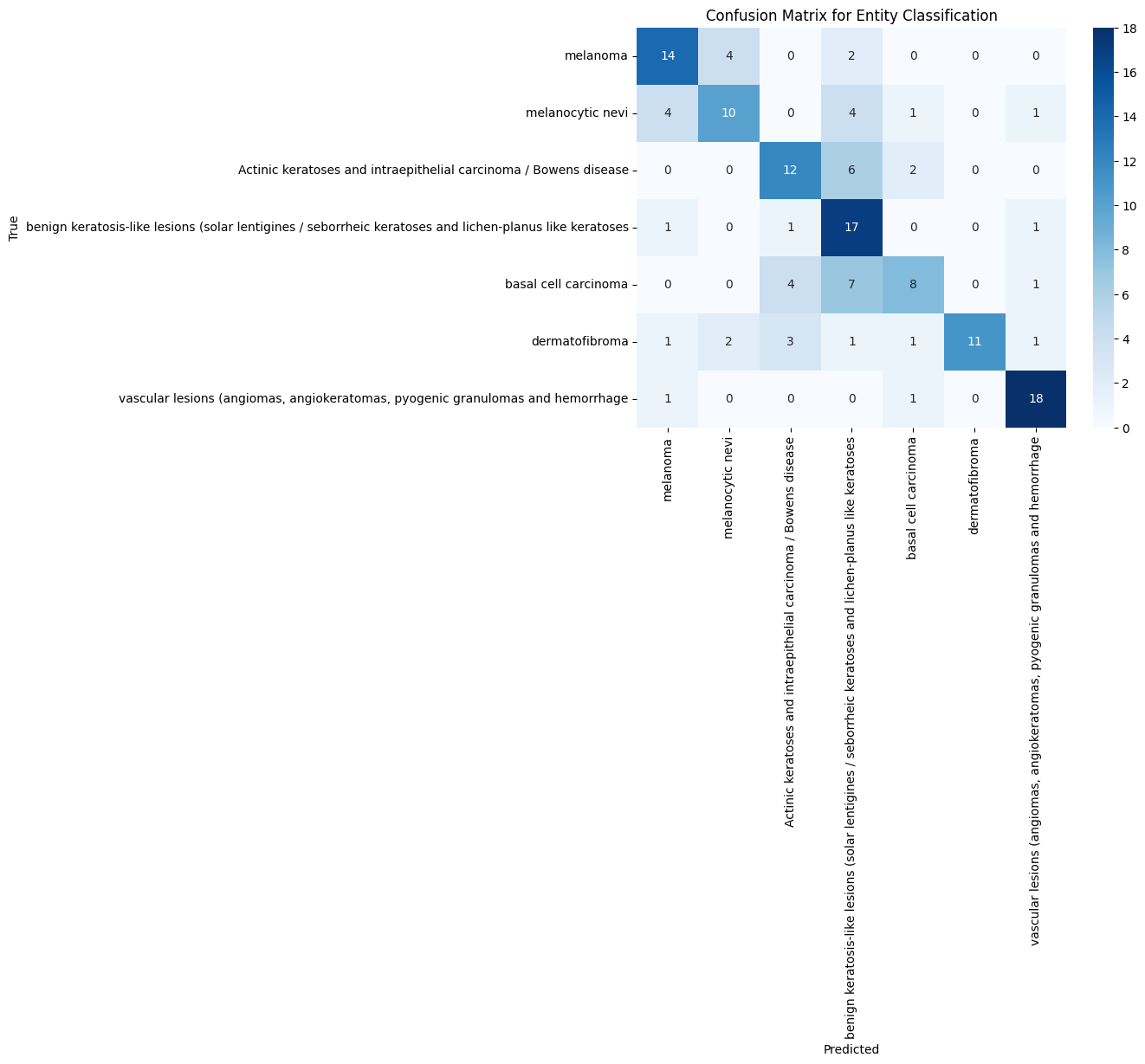

Supplement: Supplementary file 1 [file Image_1.TIFF]

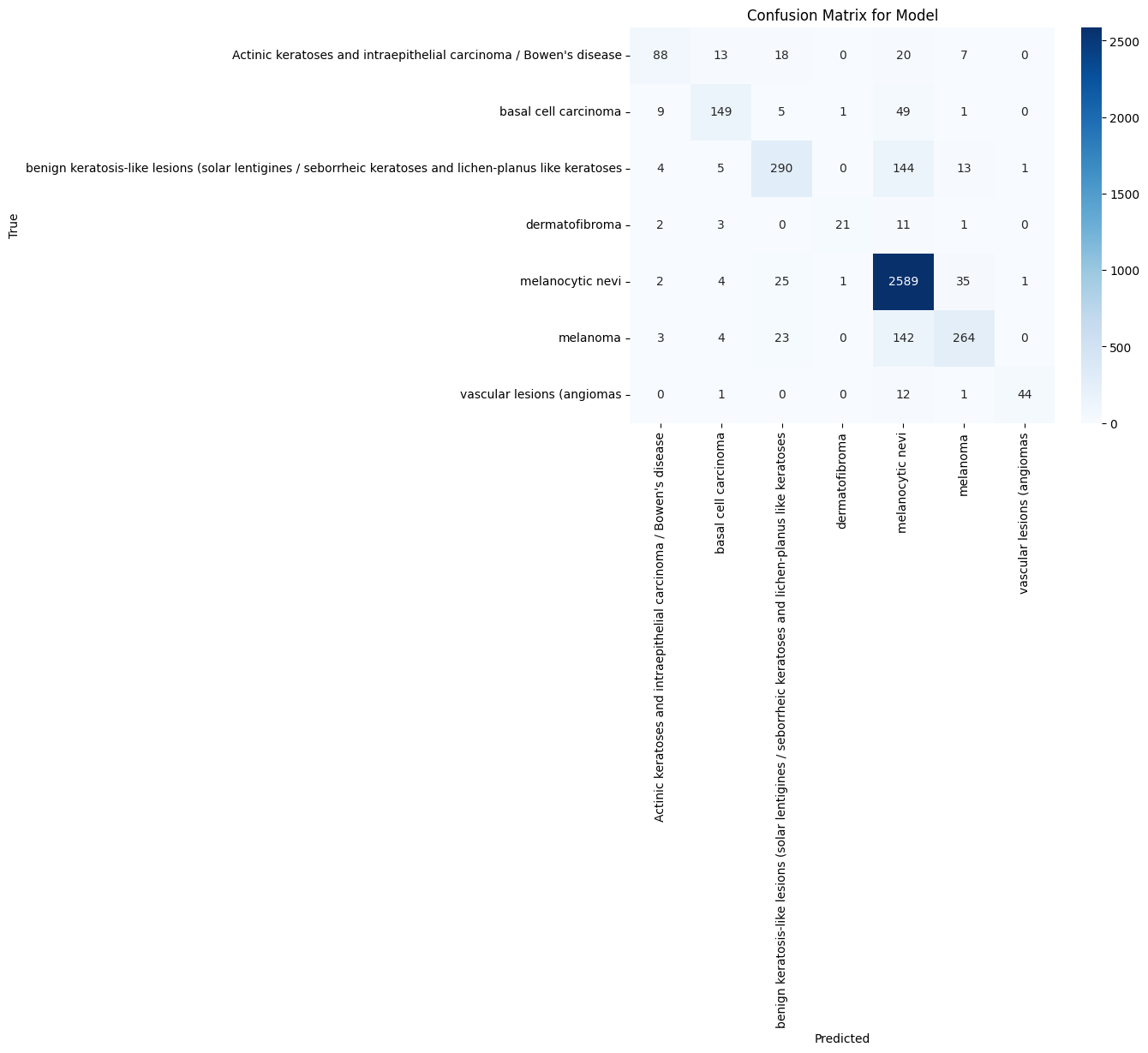

Supplement: Supplementary file 2 [file Image_2.TIFF]
